# Transient AID expression for *in situ* mutagenesis with improved cellular fitness

**DOI:** 10.1038/s41598-018-27717-2

**Published:** 2018-06-20

**Authors:** Talal Salem Al-Qaisi, Yu-Cheng Su, Steve R. Roffler

**Affiliations:** 1 Taiwan International Graduate Program in Molecular Medicine, National Yang-Ming University and Academia Sinica, Taipei, Taiwan; 2 Institute of Biomedical Sciences, Academia Sinica, Taipei, Taiwan; 3 Institute of Biochemistry and Molecular Biology, National Yang-Ming University, Taipei, Taiwan; 4 Department of Biological Science and Technology, National Chiao Tung University, Hsin-Chu, Taiwan

## Abstract

Activation induced cytidine deaminase (AID) in germinal center B cells introduces somatic DNA mutations in transcribed immunoglobulin genes to increase antibody diversity. Ectopic expression of AID coupled with selection has been successfully employed to develop proteins with desirable properties. However, this process is laborious and time consuming because many rounds of selection are typically required to isolate the target proteins. AID expression can also adversely affect cell viability due to off target mutagenesis. Here we compared stable and transient expression of AID mutants with different catalytic activities to determine conditions for maximum accumulation of mutations with minimal toxicity. We find that transient (3–5 days) expression of an AID upmutant in the presence of selection pressure could induce a high rate of mutagenesis in reporter genes without affecting cells growth and expansion. Our findings may help improve protein evolution by ectopic expression of AID and other enzymes that can induce DNA mutations.

## Introduction

Protein engineering is a powerful technique to improve protein activity, stability and other properties for industrial, diagnostic and therapeutic applications^1^. Protein engineering by rational design is very effective, but requires knowledge of the structure and function of the protein of interest^1,2^. By contrast, directed protein evolution performed by alternating rounds of random mutagenesis and selection can be employed in many cases where rational design is not feasible.

An interesting approach for autonomous mutagenesis in mammalian cells has been inspired by the germinal center reaction in which B cells overexpress the enzyme activation induced cytidine deaminase (AID), which is essential for diversification of antibody genes *in vivo*^3,4^. AID mutagenesis has been applied successfully for evolution of antibody and non-antibody proteins in both B and non-B cells^4–10^. AID mutagenesis coupled with high throughput screening in mammalian cells appears to be an ideal system for protein evolution because it avoids repetitive transfection and recloning and provides selection for proteins with better expression, activity and stability^9^. However, AID mutagenesis for protein evolution suffers from two major drawbacks; low mutation rate and genotoxicity manifested by widespread genomic instability and reduced fitness when harmful mutations accumulate in highly transcribed vital genes^11–15^. Wang *et al*. investigated the possibility of improving AID mutagenesis by increasing the specific activity of AID. Indeed, AID upmutants with increased activity were isolated, but they also led to increased genomic instability^16^ and impaired cells expansion^14^, which limits their use for protein evolution applications.

To better understand how to effectively use AID upmutants for mutagenesis in mammalian cells, we investigated *in situ* mutagenesis of a red fluorescence reporter protein by AID mutants with different enzymatic activities. We found that an AID upmutant (m7.3) generates a spike of mutagenesis shortly after expression (less than 10 days). Longer expression did not produce additional mutations in the reporter gene but reduced cell fitness and expansion. Thus, our results suggest that ideally, AID upmutants should be expressed transiently to maximize mutagenesis in target transgenes and minimize off-target toxicity.

## Results

### Somatic hypermutation reporter system

**Figure 1:**
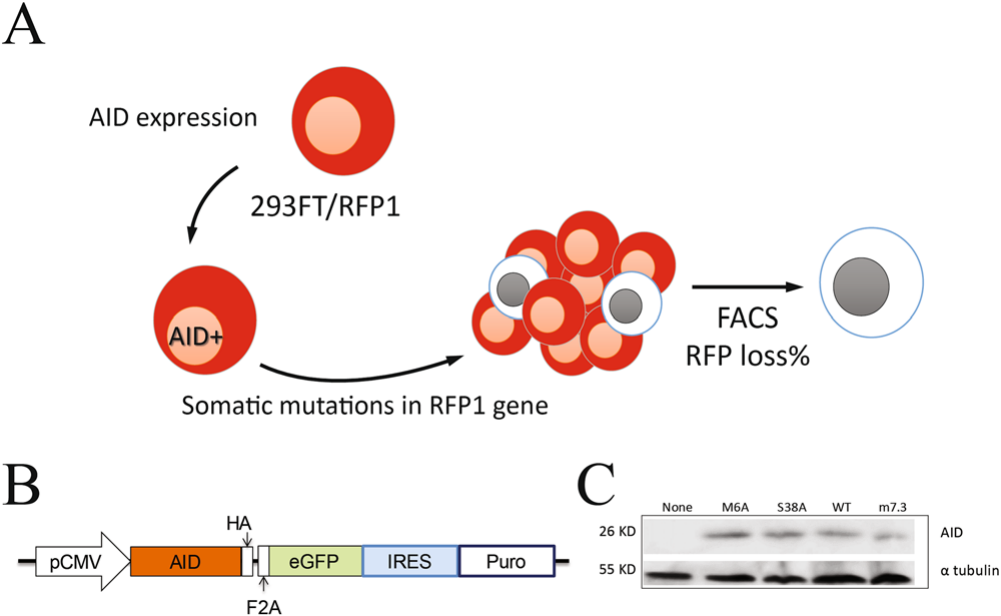
Constructs and screening system. (**A**) 293FT/RFP1 cells stably express RFP1 fluorescent protein. RFP1 florescence loss after AID expression is used to assess AID mutagenic activity. (**B**) Schematic representation of the AID expression vector. A CMV promotor is followed by a human AID or AID mutant gene which is linked to an HA tag at the C-terminus followed by furin/2 A peptide (F2A) bicistronic expression linker and an eGFP reporter gene. An internal ribosome entry site (IRES) is used for bicistronic expression of a puromycin resistance gene. (**C**) Cell lysates from 293FT/RFP1 cells expressing AID mutants were used to perform immunoblot analysis with antibodies binding to the HA tag on AID or tubulin as a cell loading control.

Because AID can mutate highly transcribed genes^17–20^, fluorescence reporter proteins are often used to monitor mutation rates^4,21^. We therefore generated a 293FT cell line that stably expresses monomeric RFP (RFP1) (Fig. 1A). Expression of AID in these cells can induce mutations in RFP gene, some of which lead to loss of RFP protein fluorescence. Analyzing the percentage of cells that lose fluorescence provides an estimate of the relative mutation rate induced by different AID mutants.

### Mutagenesis of RFP1 transgene by stably expressed AID

To investigate mutagenesis by AID, we examined either wild type AID (AID-WT) or three AID mutants; AID m7.3 (m7.3) which displays high catalytic activity^22^, AIDS38A (S38A) which displays about 30% of AID-WT activity^23^, as well as M6A which lacks AID activity^24^. AID mutants were cloned into a lentivirus expression plasmid (Fig. 1B) and recombinant lentiviral particles were used to stably infect 293FT/RFP1 cells. Expression of AID in 293FT/RFP1 cells was confirmed 3 days later by immunoblot analysis which detects the HA tag present on the recombinant AID proteins (Fig. 1C).

**Figure 2:**
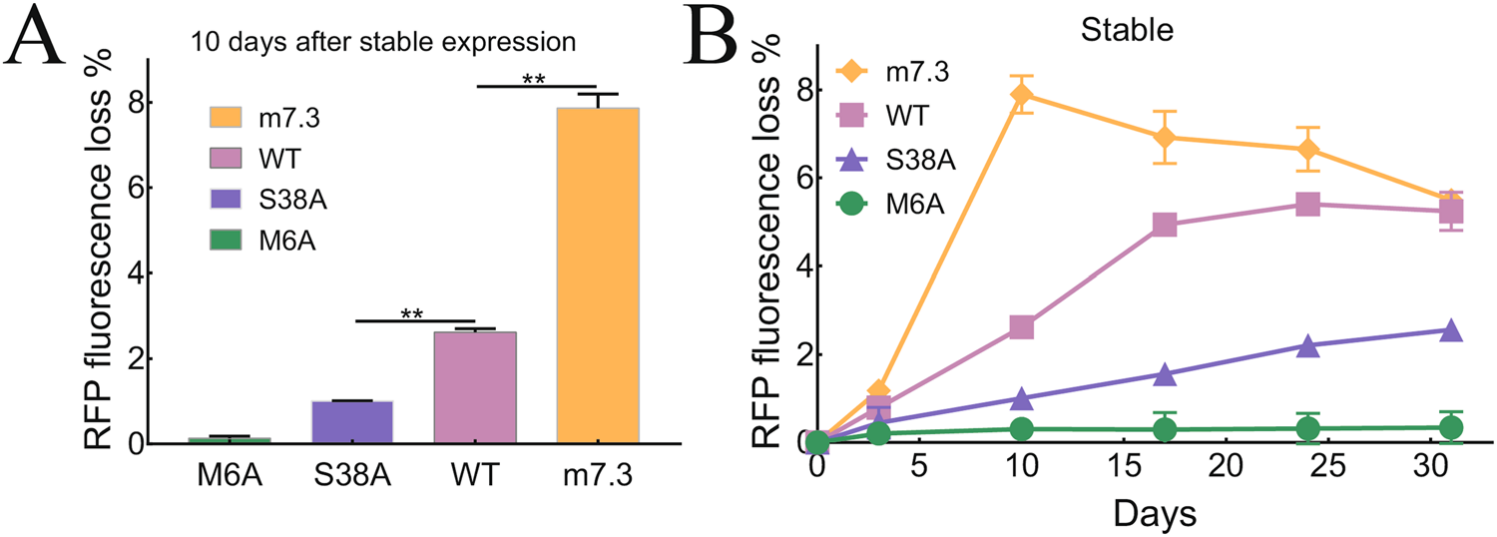
Stable AID expression induces a spike of RFP fluorescence loss. 293FT/RFP1 cells were stably transduced with AID mutants and then 10^5^ cells were analyzed for somatic hypermutation by RFP fluorescence loss using a flow cytometer (**A**) 10 days after stable expression or (**B**) periodically over one month. The results show the mean values of three replicates ± S.D. Significant differences between RFP loss after expression of S38A or m7.3 mutants compared to AID-WT are indicated; **P ≤ 0.01.

As expected, the high activity AID m7.3 mutant induced significantly more RFP negative cells on day 10 as compared to AID-WT, consistent with introduction of more mutations in the reporter gene (Fig. 2A). The low activity S38A mutant induced fewer RFP negative cells while the inactive M6A mutant produced almost no loss of RFP florescence (Fig. 2A), indicating that the percentage of RFP fluorescence loss can be used as a readout of relative mutation rates.

To study accumulation of mutations over time, we measured the percentage of RFP negative cells for one month (Fig. 2B). The m7.3 AID upmutant achieved maximum RFP florescence loss on day 10 but the frequency of RFP loss progressively decreased after this time, consistent with slower growth of cells that accumulated excessive harmful mutations. By contrast, cells that stably expressed AID-WT lost RFP fluorescence at a slower rate, which reached a plateau 24 days after infection. As expected, S38A cells accumulated RFP loss at a slower rate than AID-WT and maximum accumulation of RFP loss was not reached in 32 days.

### Transient expression of AID mutants

**Figure 3:**
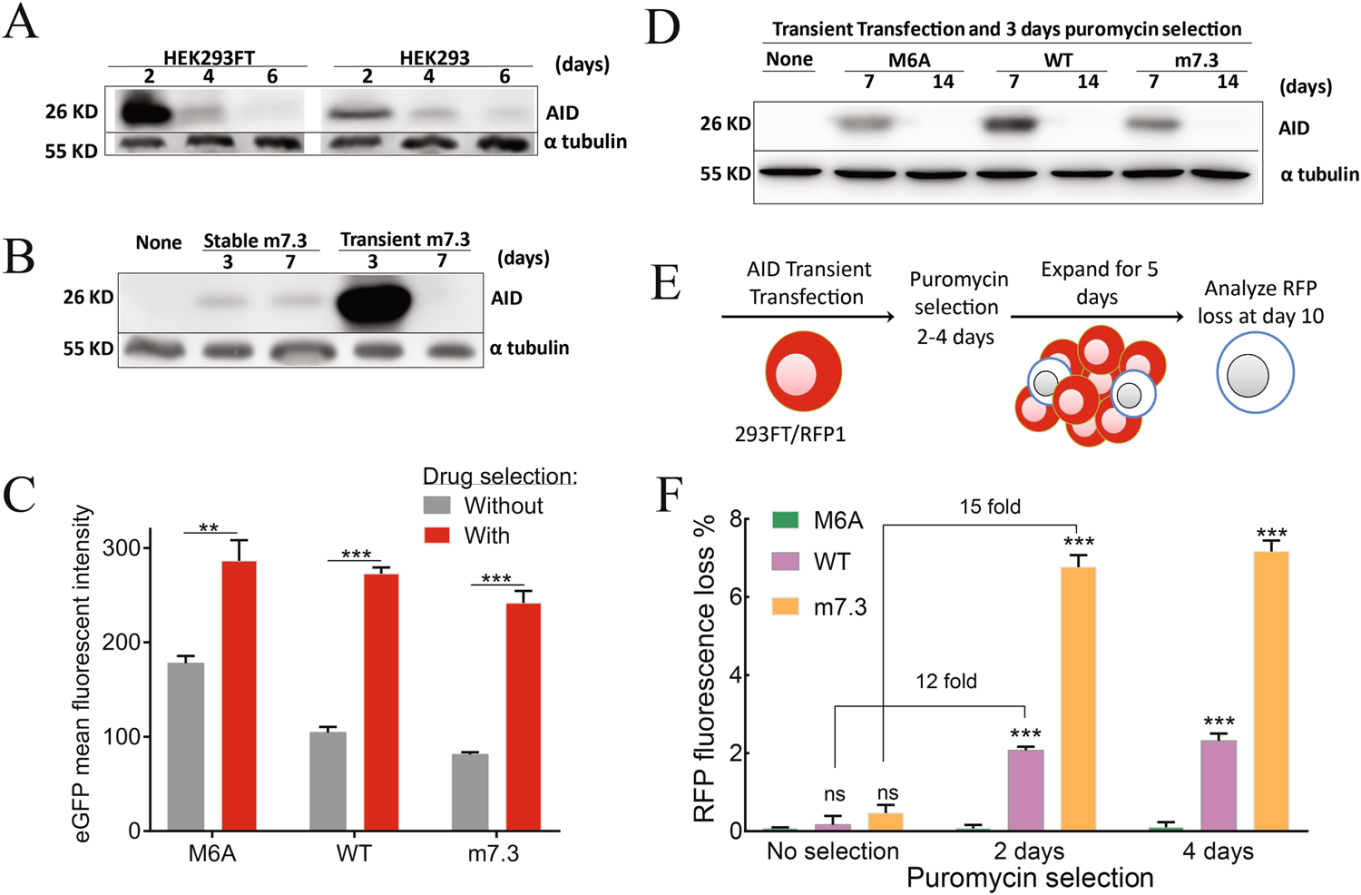
Optimization of transient transfection for efficient somatic hypermutation. (**A**) Comparison between expression of AID m7.3 in HEK293FT and HEK293 cells over time. Cells transiently transfected with m7.3 plasmid were collected every 2 days for 6 days. Cell lysates were used to perform immunoblot analysis with antibodies against the HA tag on AID or tubulin as a cell loading control. (**B**) Comparison between AID expression level after stable and transient transfection. Cells were transfected stably or transiently with m7.3 AID. Cell lysates prepared after 3 and 7 days were used to perform immunoblot analysis with antibodies binding to the HA tag on AID or tubulin as a cell loading control. (**C**) Mean fluorescence intensity of eGFP in 10^5^ cells measured at day 7 including 3 days with or without puromycin selection, n = 3; Bars, S.D. (**D**) Cells transiently transfected with AID plasmids and selected with puromycin (2 µg/mL) for 3 days. Cell lysates prepared at 7 and 14 days after transient transfection were used to perform immunoblot analysis with antibodies binding the HA tag on AID or tubulin as a cell loading control. (**E**) Reporter cells (293FT/RFP1) were transfected with different AID mutants and selected for 0, 2 or 4 days with 2 µg/mL puromycin. RFP loss was measured 10 days after transient transfection. (**F**) RFP loss with or without puromycin selection. Results show mean frequencies of RFP loss measured in 10^5^ cells at each time (n = 3). Significant differences between mean values are indicated; **P ≤ 0.01, ***P ≤ 0.001, ns, not significant.

Since the maximum mutation frequency was achieved within 10 days after stable expression of m7.3, we asked whether transient transfection with this AID upmutant is sufficient for inducing efficient somatic hypermutation. We first transiently expressed m7.3 and monitored its expression over time by Western blot analysis in HEK293FT and HEK293 cells (Fig. 3A). Although expression is very efficient after 2 days, it decreased dramatically over time probably due to plasmid dilution during cell replication. HEK293FT cells shows clear enhancement of expression after 2 days which may be attributed to replication of the plasmid (pAS3w.Ppuro), which contains the SV40 origin of replication (SV40 ori), in HEK293FT cells which express the SV40 large T antigen^25^.

We then compared expression level of m7.3 after transient and stable expression (Fig. 3B). Stable expression using lentiviral transduction produced continuous expression of AID over time, while transient transfection generated a large amount of the protein for a short time.

We then asked if we can benefit from the high efficiency of drug selection to extend AID expression by simply using short term drug selection to select cells that retain the plasmid longer. Indeed, we found that three days of drug selection was sufficient to enhance expression of the eGFP reporter by about three fold, which is expressed bicistronically with AID (Fig. 3C). Extension of AID expression was further confirmed by Western blot analysis of M6A, WT and m7.3 (Fig. 3D). AID protein was apparent at day 7 when drug selection is used, but decreased to undetectable levels by day 14 (Fig. 3D), indicating that expression is still transient, and stable transfectants were not selected by short-term exposure to puromycin.

Finally, we tested whether transient transfection with short term selection has an impact on inducing mutations in the reporter 293FT/RFP1 cells (Fig. 3E). When puromycin was not used, the mutation frequency was extremely low (Fig. 3F). By contrast, addition of puromycin for 2 or 4 days significantly enriched RFP fluorescence loss by 12 and 15 fold for AID-WT and m7.3, respectively (Fig. 3F). Enhancement of RFP loss by puromycin treatment could be due to preferential selection of cells that express AID for longer times.

### Time course and multiple AID transient transfections

**Figure 4:**
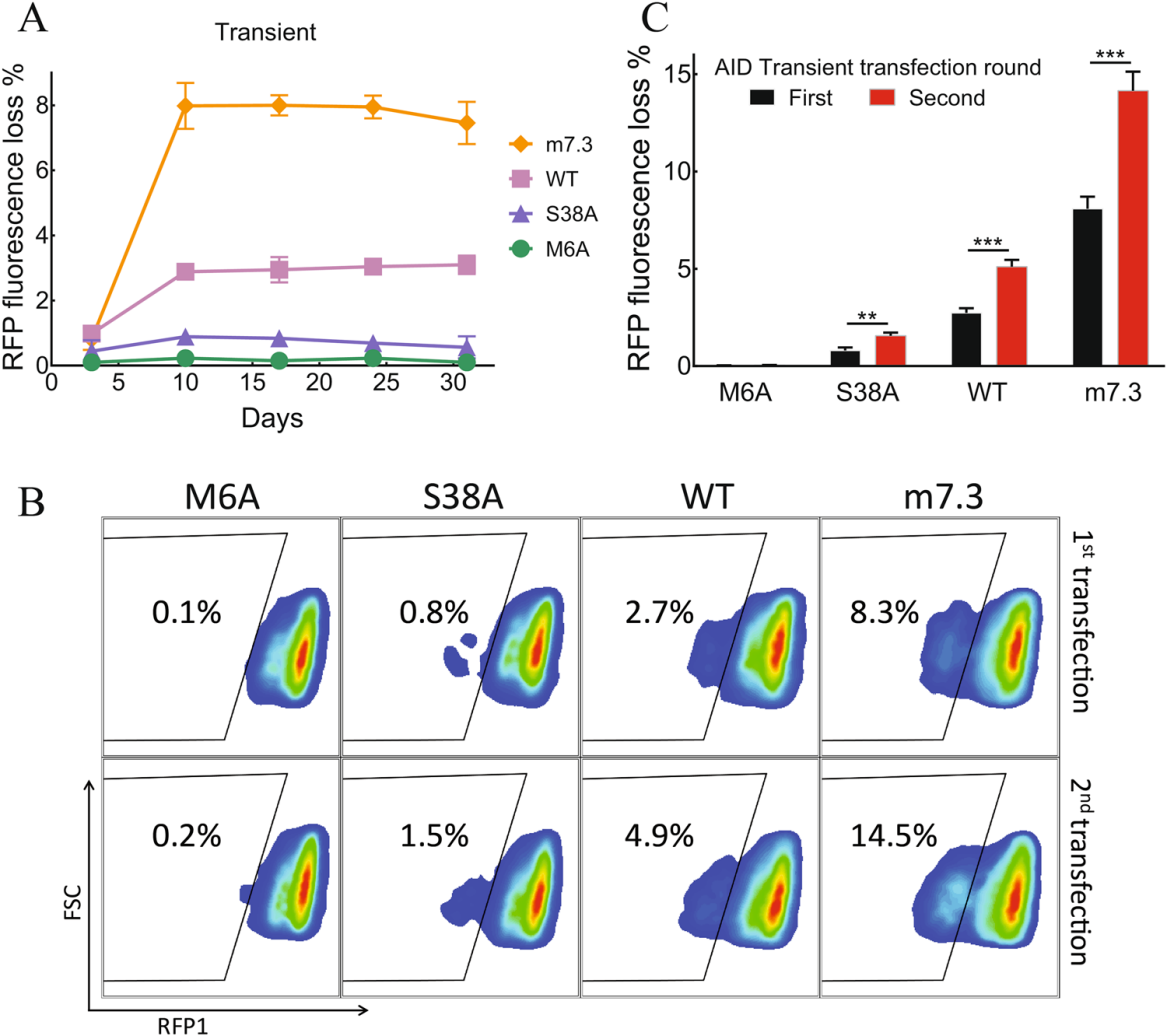
Robust RFP1 loss by transient expression of AID. (**A**) 293FT/RFP1 cells transiently transfected with plasmids encoding AID mutants, selected with puromycin for 3 days and analyzed for somatic hypermutation by RFP fluorescence loss. Results show mean values of three experiments ± SD. (**B**) Representative flow cytometry results and (**C**) analysis of accumulative RFP loss after one and two rounds of transient AID expression. Results show the mean values of three replicates ± SD. Significant differences between RFP loss after the first and the second AID transfections are indicated; **P ≤ 0.01, ***P ≤ 0.001.

Loss of RPF fluorescence was monitored for 4 weeks after transient transfection of AID mutants and selection in puromycin for 3 days. In all cases, maximum mutagenesis was achieved within 10 days after transient transfection, reaching around 3% and 8% for AID-WT and m7.3 respectively (Fig. 4A). The percentage of RFP negative cells did not change over a month follow up, consistent with the transient expression of AID and similar growth rates of the RFP positive and RFP negative cells. Limiting the time cells are exposed to AID may be important to balance between diversity generation and accumulation of deleterious mutations^26,27^.

We further examined if a second round of transient transfection can continue to produce mutations in the reporter gene, because repeated rounds of mutagenesis and selection are typically required for successful protein evolution. Indeed, the frequency of RFP negative cells significantly increased after a second round of transient transfection of AID (Fig. 4B,C), reaching around 13% of total cells with m7.3 AID.

### Transient AID expression in a DsRed reversion assay

**Figure 5:**
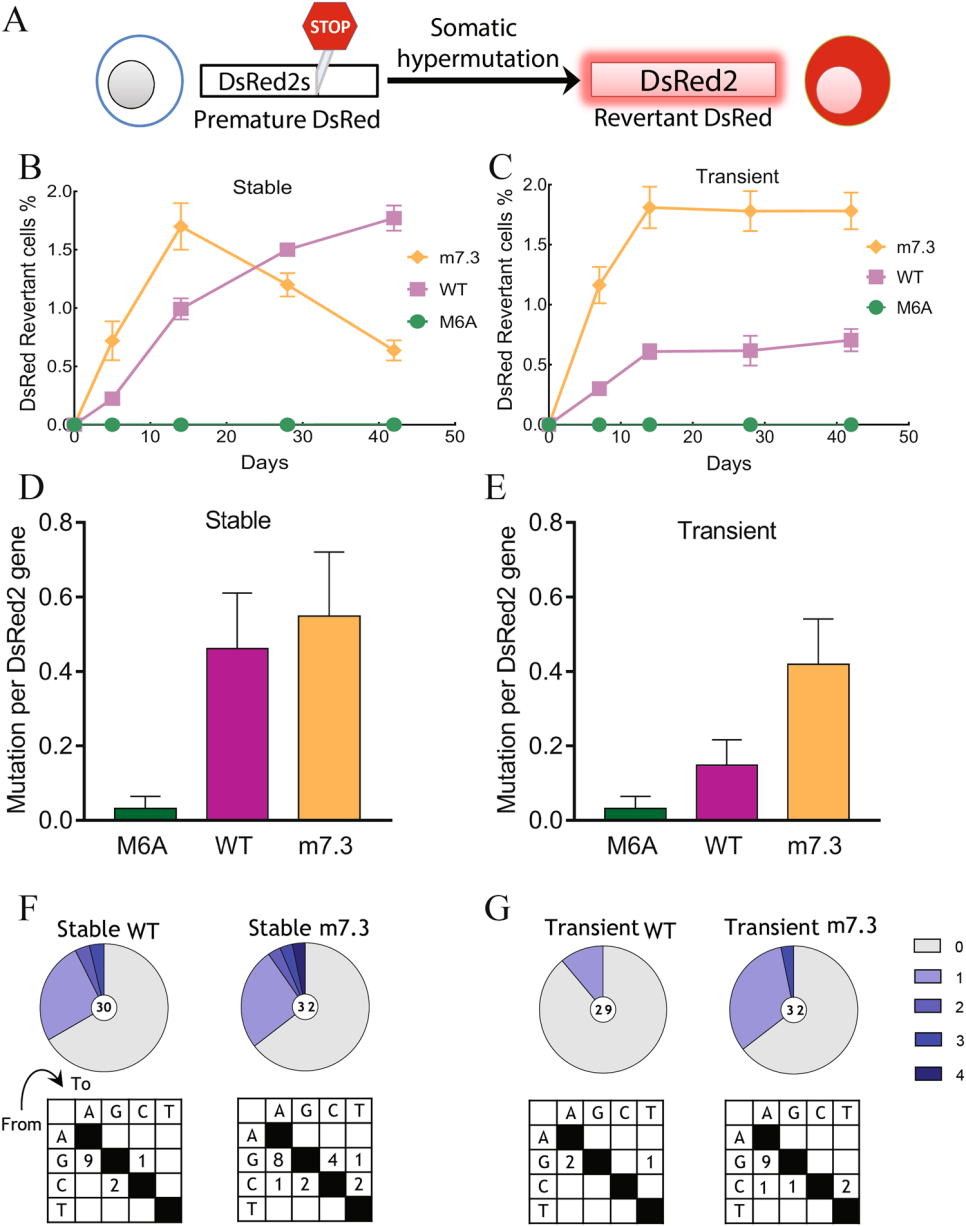
Robust DsRed reversion by transient AID expression. (**A**) 293FT/DsRed2s reporter cells stably express DsRed with a premature stop codon within an AID hotspot motif. AID expression can remove the premature stop codon and restore DsRed florescence. (**B**) 293FT/DsRed2s cells stably transduced with AID mutants (10^6^ cells) were analyzed for gain of red florescence over time. n = 3; Bars, SD. (**C**) 293FT/DsRed2s cells transiently transfected with AID mutants and selected with puromycin for 3 days (10^6^ cells) were analyzed for gain of red florescence over time. n = 3; Bars, SD. (**D**) Frequency of mutations in the DsRed2 gene obtained by PCR amplification from unsorted cells 14 days after expression of AID mutants stably or (**E**) transiently with puromycin selection. Bars, SEM. (**F**) Pie charts illustrating the number of mutations in PCR-amplified sequences from unsorted cells 14 days after expression of AID mutants stably or (**G**) transiently. The number in the middle of the pie chart indicates the total number of sequences analyzed. Below the pie charts, mutation matrix shows nucleotide substitution pattern identified from sequencing results.

To further test the usefulness of transient AID expression, we investigated reversion of a single fatal base pair in the red fluorescent protein (DsRed2) gene. We introduced a pre-mature stop codon within an AID hotspot motif, which renders the DsRed2 gene non-functional (Fig. 5A). Emergence and retention of DsRed2 fluorescence was studied over time after mutagenesis by AID-WT and m7.3 by either stable or transient transfection protocols. Stable expression of m7.3 (Fig. 5B) induced rapid emergence of red fluorescence, which reached a maximum frequency of 1.7% after two weeks and then gradually decreased over time. Stable expression of AID-WT required about 42 days to accumulate a similar number of DsRed2 positive cells. Transient transfection of m7.3 also produced about 1.8% DsRed2 positive cells after two weeks (Fig. 5C), but in contrast to stable expression of m7.3, there was no obvious outgrowth of DsRed2 negative cells at later times, indicating less damage to cells.

Furthermore, the reporter DsRed2 reporter system was validated by sequence analysis, which revealed that mutation load of the unsorted cells after 14 days of stable or transient expression of AID mutants correlates with the percentage of revertant cells in the DsRed revertant system (Fig. 5D,E). Mutations in all groups were predominantly transition mutations and mainly G to A mutations (Fig. 5F,G).

### Toxicity of Stable AID expression

**Figure 6:**
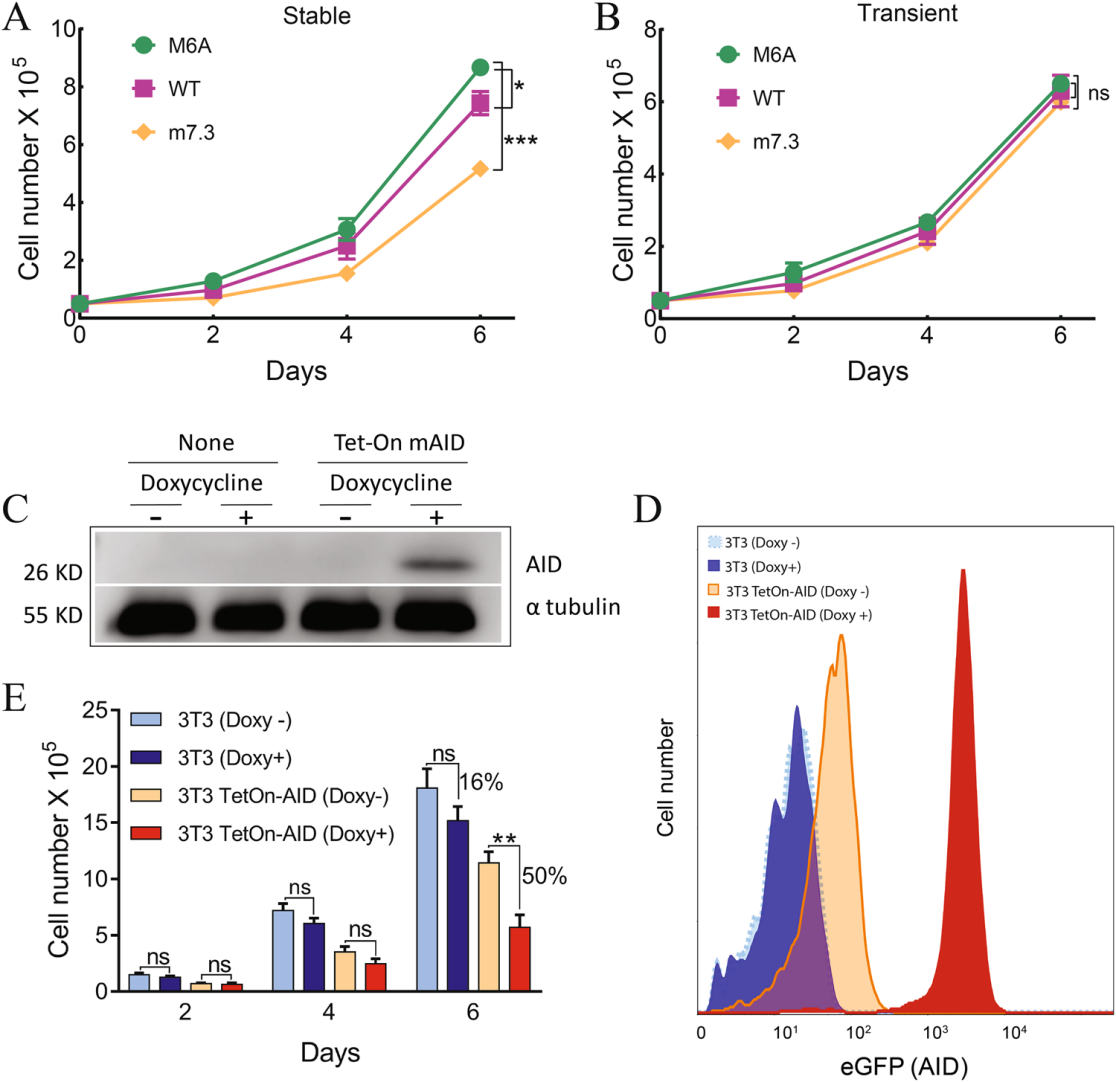
Transient expression of AID reduces its adverse effect on cell growth. Growth curve of HEK293FT cells (**A**) virally transduced with AID mutants or (**B**) transiently transfected with AID expressing plasmids. Results show mean values ± SD (n = 3). (**C**) Western blots show AID expression in cell extracts from 3T3 and 3T3 TetOn-AID cells after 4 days of culturing the cells with or without 0.5 µg/mL doxycycline; tubulin was used as loading control. (**D**) AID induction as confirmed by bicistronic GFP expression in 10^5^ cells of each 3T3 and 3T3 TetOn-AID cells after 4 days of culturing the cells with or without 0.5 µg/mL doxycycline. (**E**) Growth curve of 3T3 and 3T3 TetOn-AID cells with or without 0.5 µg/mL for 6 days. Results show mean values ± SD (n = 3). Significant differences between growth curves are indicated; *p ≤ 0.05; **p ≤ 0.01; ***p ≤ 0.001. ns, not significant.

Both transient and stable expression of AID can effectively introduce mutations into reporter genes, but stable expression of AID appeared to adversely affect cell viability, in agreement with studies demonstrating toxicity induced by uncontrolled expression of AID^11–15^. To confirm the adverse effect of AID expression on cell expansion, we stably or transiently expressed M6A, AID-WT or m7.3 in 293FT cells, seeded similar number of cells and counted viable cells every 2 days for 6 days. Indeed, we found that stable (Fig. 6A) but not transient (Fig. 6B) expression of both AID-WT and m7.3 significantly decreased cell growth as compared to the control group expressing the inactive M6A mutant.

Toxicity of AID was further confirmed in 3T3/TetOn-AID, a mouse cell line stably expressing a tetracycline-inducible AID expression cassette^10^. Controllable induction of AID expression was confirmed by Western blot analysis (Fig. 6C), and eGFP reporter (Fig. 6D). Continuous induction of AID expression in these cells for 6 days shows 50% growth inhibition compared to cells cultured in doxycycline-free medium. Growth retardation can be partially attributed to doxycycline itself, but this is a minor effect because parental 3T3 cells are only inhibited by 16% after exposure to the same dose of doxycycline for the same time period.

## Discussion

We report a simple and reproducible protocol to generate somatic hypermutations in transgenes with reduced toxicity using the m7.3 hyperactive AID mutant. The protocol relies on transient bicistronic expression of m7.3 and a puromycin resistance gene combined with short-term puromycin selection. The devised protocol was based on the finding that the mutation rate declines quickly with concurrent decline of cell proliferation rate after stable expression of m7.3. We predicted that limiting the expression time of m7.3 could reduce its adverse effects. Indeed, transient expression of m7.3 for 3–5 days under puromycin selection was an efficient to introduce mutations in a reporter gene with almost no adverse effects on cell fitness. We confirmed that mutagenesis can be controlled by puromycin selection, mutagenesis is very efficient when AID is introduced under selection and removal of puromycin greatly reduced AID level and mutation rate. Taken together, our study reports a simple way to diversify highly transcribed genes with reduced toxicity and without the need for library construction, protein engineering or recombinant antibody production.

Protein evolution requires repetitive cycles of introducing mutations and selection^28^. The efficiency of mutagenesis is a key factor for fast and efficient protein evolution^29,30^. *In vitro* mutagenesis by error prone PCR or DNA shuffling generates high mutation rates, but requires repeated cloning and transfection. *In vivo* mutagenesis methods require minimal intervention because selection and mutagenesis are coupled in the same system. Most *in vivo* mutagenesis methods suffer from low mutation rates and increased off-target mutagenesis^31^. Therefore, there is a need for an *in vivo* mutagenesis systems which can diversify a chosen gene of interest with a high frequency.

Natural somatic hypermutation is an efficient system for antibody diversification. *In vivo*, about one point mutation is generated in an immunoglobulin gene per cell division^32^. This requires the enzyme activation-induced cytidine deaminase (AID) which deaminates deoxycytidine residues to uracil in actively transcribed genes^33–36^. Uracil is mutagenic when paired with guanosine in DNA, since dU mimics dT during replication, and the U:G mismatch triggers error-prone DNA repair in B cells. Thus, AID introduces somatic hypermutation (SHM) by converting dC to dU^37–40^. The power of SHM for protein evolution was first shown in a cell line that constitutively expresses AID (Ramos cells); Cumbers *et al*. were able to evolve endogenous IgM from unknown specificity to selectively bind streptavidin with nanomolar affinity after 19 rounds of FACS sorting^41^. Since that time, several studies reported using hypermutating cell lines like Ramos and DT40 for protein evolution^4,42,43^. Interestingly, ectopic expression of AID in non-B cells also turns on somatic hypermutation and diversifies highly transcribed transgenes^18^. Successful antibody evolution was reported after ectopic AID expression in human lung carcinoma^44^, HEK293^10,45–47^ and CHO^48^ cells.

A major drawback of inducing mutations by endogenous AID is that mutations are still far below that observed *in vivo*^20,49,50^. Ectopic overexpression of AID can partially solve this problem, but it increases off-target mutagenesis of highly expressed genes and transgenes such as AID itself^20,51^. Hypermutation rate *in vivo* has been estimated to be approximately 10^−3^ mutations per base pair per generation; this frequency seems to be optimal as a lower frequency might generate poor diversity, whereas a higher mutation rate might cause increased cellular toxicity^52–57^.

To improve the efficiency of AID mutagenesis, Wang *et al*. generated human AID upmutants with improved activity, but unfortunately the increase in catalytic activity was accompanied with increased adverse effects on DNA instability and cells fitness^22^. However, the increased genomic instability associated with the AID upmutants was assayed by stable expression of AID upmutants in B cells^22^. By contrast, AID is transiently expressed for short periods in B cells during antibody affinity maturation^26,58^. Therefore, we decided to test transient expression of AID to induce mutations in transgenes with reduced cellular toxicity.

Transient transfection of AID for 3–5 days under puromycin selection was ideal to generate SHM with reduced toxicity. By contrast, transient transfection without any selection pressure produced extremely low mutation frequency. Since the puromycin resistance gene is a highly transcribed transgene, it has a chance to be targeted by AID. We used the shortest possible time for drug selection to avoid killing the cells that accumulate more mutations in the puromycin-resistance gene, which are probably the cells with better SHM activity. Puromycin was useful for pulse selection because it can kill the cells that did not express the gene in as short as 1 or 2 days using low drug concentration^59–61^.

An interesting application of the findings in this study is to mimic the natural cancer progression and metastasis mechanisms. There is a mounting evidence that mutagenesis by AID and other closely related deaminases play important roles in cancer evolution and metastasis^62–64^. It was hypothesized that transient but not constitutive mutagenesis facilitates cancer progression^12,65^. Indeed our data support this hypothesis because it seems that continuous mutagenesis might have eventual adverse effects on cancer fitness due to continuous accumulation of mutations, which might adversely affect cancer growth. This concept might be useful for researchers interested in studying cancer pathogenesis. For example, a cytidine deaminase can be expressed for a short time and cells with interesting phenotype can be selected. This could be a useful approach to allow researchers to isolate customized cancer clones based on the selection criteria they want.

In this study, we provide a simple solution for fast, controllable, low toxicity mutagenesis of highly transcribed genes by using transient hyperactive AID expression. AID gene transfection generates a library of mutants for the gene of interest inside the cells without the need for using error-prone PCR or DNA shuffling. Proteins with improved properties can be isolated, propagated and further mutagenized by additional rounds of mutagenesis by AID upmutant. Besides selection for improved protein function, mutants can also be selected for other desired properties such as solubility, expression, and post-translational modification.

## Material and Methods

### Cell lines and culture conditions

Ramos human Burkitt’s lymphoma B cells (CRL-1596, American Type Culture Collection, Manassas, VA, USA) were grown in RPMI 1640 medium supplemented with 2 mM L-glutamine and 10% heat-inactivated fetal bovine serum (HyClone, Logan, UT, USA). BALB/3T3 fibroblasts and HEK293 cells were obtained from ATCC (Manassas, VA, USA). HEK293FT human embryonic kidney cells (Thermo Fisher Scientific, San Jose, CA, USA). BALB/3T3 fibroblasts, HEK293 and HEK293FT were maintained in Dulbecco’s modified Eagle’s medium (Sigma, Saint Louis, MO, USA) supplemented with 10% fetal calf serum, 100 μg/mL streptomycin and 100 U/ml penicillin grown in a humidified incubator at 37 °C in 5% CO_2_ and 95% air. 3T3/TetOn-AID cells^10^ were maintained in Dulbecco’s modified Eagle’s medium supplemented with 10% dialyzed fetal calf serum, 100 μg/mL streptomycin and 100 U/ml penicillin grown in a humidified incubator at 37 °C in 5% CO_2_ and 95% air.

### Generation of Reporter cells

293FT/mRFP1 and 293FT/DsRed2s reporter cells were generated to assess mutation rates. The mRFP1 gene was amplified from mRFP1 pRSET B plasmid which was a kind gift from Dr. R. Y. Tsien (University of California, San Diego, USA). mRFP1 was amplified by PCR with a 5′ primer 5′-ctagcggcgcgccgccatggtgagcaagggcgagg-3′ that encodes an *AscI* restriction site and a 3′ primer 5′-gagaggttaacttaggcgccggtggagtggcggc-3′ that encodes a *Hpa*I site. mRFP was ligated into the backbone of pLKO_AS3w-Hygro (National RNAi Core Facility, Institute of Molecular Biology/Genomic Research Center, Academia Sinica, Taiwan) to generate pLKO_AS3w-RFP1 lentiviral vector for mammalian cell expression.

The DsRed2s gene was amplified by PCR from the pLHCX-DsRed2s.hyg plasmid^10^ using the forward primer 5′-ggaggtggcgcgccgccaccatggcctcctccgag-3′ and reverse primer 5′ggtggagtttaaacggtcaactgtaatcttggccacctg-3′and then inserted *into AscI* and *PmeI* sites downstream of the CMV promoter in pLKO_AS3w-Hygro to generate pLKO_AS3w- DsRed2s lentiviral vector.

Lentiviral plasmids encoding mRFP1 and DsRed2s were co-transfected with pCMVΔR8.91 packaging plasmid and pMD.G VSV-G envelope plasmid in HEK293FT cells to produce lentiviral particles^10^. Two days later, the culture medium was filtered, mixed with 8 μg/ml polybrene, and incubated with HEK293FT cells at a multiplicity of infection (MOI) of 0.1 to ensure single copy of reporter gene per cell. Stable cell lines were selected with 200 μg/ml hygromycin for two weeks.

### AID gene amplification

Ramos cells (1 × 10^6^) were lysed with 1 ml Super RNApure reagent and RNA was extracted according to the manufacturer’s instructions (Genesis Biotech Inc., Taipei, Taiwan). Five micrograms of RNA were reverse transcribed using the Superscript III first-strand synthesis system (Thermo Fisher Scientific). Phusion high fidelity DNA polymerase was used to amplify the AID gene using primers 5′ ctagtgctagcgccaccatggacagcctcttgatgaac-3′ and 5′-cgtgagttaacacctccaagtcccaaa-gtacgaa-atgc-3′.

### AID mutant construction

PCR products were cloned by Zero Blunt PCR cloning kit (Thermo Fisher Scientific) and sequenced using standard M13 forward and reverse primers. Multiple site mutagenesis was performed as described^66^ in the wild-type human AID gene to generate the AID m7.3 mutant (K10E, T82I, and E156G) with increased catalytic activity^22^. Site directed mutagenesis using QuikChange™ Site-Directed Mutagenesis Kit (Stratagene, La Jolla, CA, USA) was used to generate the AID S38A mutant with compromised activity^67^ and AID-M6A with total loss of activity^24^. Wild type AID and mutants AID genes were cloned into a lentiviral vector pAS3w.Ppuro^10^ by the *HpaI* and *NheI* restriction sites (New England BioLabs, Ipswich, MA, USA). An HA tag epitope (YPYDVPDYA) was fused to the C-terminus of AID and AID mutants to allow detection by immunoblot analysis.

### Stable expression of AID

Lentiviral plasmids encoding AID mutants were used to generate recombinant lentiviral particles. Virus packaging was done by co-transfection of 7.5 μg of AID, m7.3, S38A, or M6A with pCMVΔR8.91 (6.75 μg) and pMD.G (0.75 μg) using TransIT-LT1 transfection reagent (Mirus Bio, Madison, WI, USA) (45 μL) in HEK293FT cells grown in a 10 cm culture dish (90% confluency). After 48 h, lentiviral particles were suspended in culture medium containing 8 μg/mL polybrene and filtered through a 0.45 μm filter. 293FT/mRFP1 or 293FT/DsRed2s cells were seeded in 6-well plates (1 × 10^5^ cells/well) one day before viral infection. Lentivirus containing medium was added to the plate and then centrifuged for 1.5 h (500 g, 32 °C). The cells were selected in puromycin (2 μg/mL) for 4 days to generate stable cell lines.

### Transient expression of AID

Transient transfection was performed using TransIT-LT1 Transfection Reagent (Mirus Bio) according to the manufacturer’s instructions. Briefly, 293FT, 293FT/RFP1 or 293FT/DsRed2s cells were seeded in 6-well plates in DMEM containing 10% FBS without antibiotics one day before transfection, such that they were 70–90% confluent at the time of transfection. On the day of transfection, 2.5 µg of AID lentiviral vectors and 7.5 µL of TransIT-LT1 were combined in 500 µL of Opti-MEM Reduced Serum Medium (Thermo Fisher Scientific). The mixture was incubated for an additional 25 min at room temperature and then added to each well, and the cells were incubated at 37 °C in a CO_2_ incubator for 24 hours. Cells were then refreshed with DMEM containing 10% FBS for one day, and then selected with 2 µg/mL puromycin for 2 or 4 days.

### Immunoblotting

To measure AID protein levels, 5 × 10^6^ cells were lysed in 0.5 mL RIPA buffer (1% NP-40, 150 mM NaCl, 0.5% sodium deoxycholate, 0.1% SDS, 50 mM Tris, pH 8.0) for 1 h at 4 °C. Fifty μg of total protein from the clarified lysates were separated on a 12.5% reducing SDS-PAGE, transferred to nitrocellulose paper and sequentially stained with biotinylated goat anti-HA antibody (Vector Laboratories, CA, USA) or rabbit anti-tubulin α antibody (NeoMarkers, CA, USA) followed by streptavidin-HRP and goat anti-rabbit Ig-HRP, respectively (Jackson ImmunoResearch Laboratories, PA, USA). Bands were visualized by ECL detection (Thermo Fisher Scientific) and analyzed with a LAS-3000 Mini Fujifilm imaging system (FujiFilm, Tokyo, Japan).

### Flow cytometry

The percentage of RFP1 negative or DsRed2 positive cells was determined by flow cytometry. Cells were washed with phosphate buffered saline (PBS) and then incubated with 1 µM Sytox Blue viability dye (Thermo Fisher Scientific) in PBS containing 0.05% bovine serum albumin (BSA) at room temperature for 30 minutes before measurement on a SRII flow cytometry (Becton Dickinson, Mountain View, CA, USA), with analysis by FlowJo software (Tree Star, San Carlos, CA, USA).

### Cell proliferation assay

Two days after transient or stable transfection of HEK293FT cells with AID mutants, cells were selected in medium containing 2 µg/mL puromycin for 4 days, and refreshed with puromycin-free media for 1 day. Similar numbers of cells were seeded in 6 well plates and viable cells were counted every 2 days by trypan blue dye exclusion using a Countess Automated Cell Counter (Thermo Fisher Scientific).

Stably infected 3T3/TetOn-AID was reported previously. Cells were seeded in 6 cm plates with or without 0.5 µg/mL doxycycline, and viable cells are counted every 2 days for 6 days.

### Sequencing

Mutations in the DsRed2 gene were characterized by sequencing of genomic DNA that was PCR-amplified from 10^6^ cells 2 weeks after transfection. The PCR product was ligated to zero-blut vector (Thermo Fisher Scientific) and sequenced to confirm the mutations.
